# Human-Biomonitoring für Europa (HBM4EU) – erste Einblicke in die Ergebnisse der Initiative

**DOI:** 10.1007/s00103-022-03578-z

**Published:** 2022-08-23

**Authors:** Philipp Weise, Petra Apel, Marike Kolossa-Gehring

**Affiliations:** FG II 1.2 Toxikologie, gesundheitsbezogene Umweltbeobachtung, Umweltbundesamt, Corrensplatz 1, 14195 Berlin, Deutschland

**Keywords:** HBM, Innere Schadstoffbelastung, Exposition, Chemikalienpolitik, Risikobewertung, HBM, Internal pollution, Exposure, Chemical policy, Risk assessment

## Abstract

Beim Human-Biomonitoring wird die innere Schadstoffbelastung des Menschen aus verschiedenen Quellen wie Nahrung, Alltagsgegenständen oder Atemluft erfasst, indem z. B. Blut und Urin analysiert werden. Um das Human-Biomonitoring in Europa zu fördern und zu koordinieren, wurde 2017 das Projekt „Human-Biomonitoring für Europa“ (HBM4EU) begonnen, an dem sich 30 Länder, die Europäische Umweltagentur und die Europäische Kommission beteiligt haben. Im Juni 2022 wurde das Projekt abgeschlossen.

Vergleichbare und zuverlässige Belastungsdaten konnten für eine breite Palette von Umweltchemikalien erfasst und einheitlich bewertet werden. Weitere wichtige Erfolge der Initiative waren die Etablierung eines Kontrollprogramms zur Qualitätssicherung, ein Konzept zur Vereinheitlichung zukünftiger HBM-Studien, eine gemeinsame Strategie zur Ableitung von gesundheitsbezogenen Beurteilungswerten (HBM Guidance Values – HBM-GVs) und die Einrichtung nationaler Gremien. Die gewonnenen Belastungsdaten sind über die Informationsplattform für die Überwachung von Chemikalien (IPCHEM) und das EU HBM-Dashboard zugänglich. Publikationen sind über die HBM4EU-Onlinebibliothek frei verfügbar.

Insgesamt zeigen die Ergebnisse, dass die Belastungen der EU-Bevölkerung für viele Chemikalien wie etwa Phthalate und perfluorierte Alkylsubstanzen (PFAS) zu hoch sind und weiterhin Handlungsbedarf seitens der Politik besteht. Das im Projekt HBM4EU generierte Wissen kann die politischen Entscheidungsträger:innen bei der Verbesserung der Chemikalien‑, Umwelt- und Gesundheitspolitik unterstützen.

## Abstract (en)

Human biomonitoring measures the internal exposure of humans to chemicals from various sources, such as food, everyday objects, or the air we breathe, by analyzing blood and urine, for example. To promote and coordinate human biomonitoring in Europe, the European Human Biomonitoring Initiative (HBM4EU) was launched in 2017 and involves 30 countries, the European Environment Agency, and the European Commission. The project was completed in June 2022.

Comparable and reliable exposure data were collected and consistently assessed for a wide range of environmental chemicals. Other important achievements of the initiative were the establishment of a quality assurance program, a concept for standardizing future HBM studies, a common strategy for deriving health-based guidance values (HBM-GVs), and the establishment of national committees in the partner countries. The exposure data generated are accessible via the Information Platform for Chemical Monitoring (IPCHEM) and the EU HBM Dashboard. Publications are freely available through the HBM4EU online library.

Overall, the results show that exposures of the EU population to many chemicals are too high, such as phthalates and perfluorinated alkyl substances (PFAS), and that policy action is still needed. The knowledge generated in the HBM4EU project can support policymakers in improving chemical, environment, and health policies.

Im Juni 2022 wurde die Initiative Human-Biomonitoring für Europa (HBM4EU) abgeschlossen. Dieses gemeinsame Projekt von 30 Ländern, der Europäischen Umweltagentur und der Europäischen Kommission, das im Rahmen von Horizon 2020, dem EU-Programm für Forschung und Innovation, kofinanziert wurde, lief seit 2017 und wurde vom Umweltbundesamt koordiniert.

Die Initiative diente dazu, das Human-Biomonitoring (HBM) in Europa zu fördern und zu koordinieren. Dabei wird beim Belastungsmonitoring die Schadstoffbelastung bestimmter Bevölkerungsgruppen (Allgemeinbevölkerung oder bestimmte Berufsgruppen) aus verschiedensten Quellen, wie z. B. aus der Nahrung, Alltagsgegenständen oder der Atemluft, durch Analysen von z. B. Blut oder Urin erfasst. HBM liefert somit Erkenntnisse über die tatsächliche Exposition der Bürger:innen mit Schadstoffen und erlaubt Rückschlüsse auf mögliche gesundheitliche Auswirkungen. Das generierte Wissen über die tatsächlichen Belastungen unterstützt letztlich die politischen Entscheidungsträger:innen bei der Verbesserung der Chemikalien‑, Umwelt- und Gesundheitspolitik [1].

Mit HBM4EU wurde ein innovatives Forschungsnetz geschaffen, das den Grundstein für die langfristige Etablierung eines Human-Biomonitoring-Systems in Europa gelegt hat. Indem die Erwartungen und Bedürfnisse der politischen Entscheidungsträger:innen auf nationaler und EU-Ebene sowie aller Interessengruppen von Anfang an berücksichtigt wurden, war es möglich, ein leistungsfähiges HBM-Netzwerk aufzubauen, das alle wichtigen Akteure im Bereich des HBM umfasst und einen engen Dialog zwischen der wissenschaftlichen Gemeinschaft und den EU-Agenturen ermöglicht.

Dieser Artikel gibt eine Übersicht über die Ergebnisse des Projekts.

## Priorisierung der zu untersuchenden Substanzen

In einem ersten Schritt wurde unter Einbeziehung sämtlicher Interessengruppen eine Strategie zur Priorisierung der in HBM4EU zu untersuchenden Substanzen/Substanzgruppen entwickelt [2]. Die Auswahl der finalen 18 Substanzen/Substanzgruppen^1^ erfolgte in 2 Priorisierungsrunden.

Bei den priorisierten Substanzen/Substanzgruppen handelt es sich um:

- Acrylamid,
- Cadmium,
- Quecksilber,
- Aniline,
- chemische Mischungen,
- Mykotoxine,
- aprotische Lösemittel,
- Chrom VI,
- perfluorierte Alkylsubstanzen (PFAS),
- Arsen,
- neue Chemikalien,
- Pestizide,
- Benzophenone,
- Flammschutzmittel,
- Phthalate und Hexamoll® DINCH,
- Bisphenole,
- Blei,
- polyzyklische aromatische Kohlenwasserstoffe (PAK, engl.: PAHs).

## Harmonisierung auf EU-Ebene und Einrichtung nationaler Gremien

Ein grundlegendes Ziel der Initiative HBM4EU war es, auf EU-Ebene vergleichbare und zuverlässige Expositionsdaten für eine breite Palette von Umweltchemikalien zu erfassen und diese einheitlich zu bewerten, damit europäische Institutionen auf dieser Basis effektive Maßnahmen zum Schutz der menschlichen Gesundheit und der Umwelt treffen können.

Ein wichtiger Erfolg des Projekts ist hierbei die Etablierung eines Qualitätssicherungs‑/Qualitäts(QA/QC-)Kontrollprogramms und des Netzwerks europäischer Laboratorien, welche vergleichbare Analysedaten von hoher Qualität liefern [3]. Das QA/QC-Programm ist ein wichtiger Schritt zum Aufbau eines nachhaltigen europäischen Netzwerks von Human-Biomonitoring(HBM-)Laboratorien.

Um zukünftige HBM-Studien zu vereinheitlichen und Daten vergleichbar zu erheben, wurde ein Konzept für die Planung und Durchführung von HBM-Studien [4] sowie zur Entwicklung von Fragebögen [5] erarbeitet.

Darüber hinaus wurde eine Strategie zur Ableitung von gesundheitsbezogenen Beurteilungswerten, sogenannten HBM Guidance Values (HBM-GVs), gemeinsam vereinbart [6], so dass nun die Auswertung erhobener Daten harmonisiert erfolgen kann. Die HBM-GVs können zur Bewertung der HBM-Ergebnisse direkt mit den Messwerten verglichen werden und beziehen sich auf die Allgemeinbevölkerung oder auch auf beruflich exponierte Erwachsene. Die abgeleiteten HBM-GVs wurden zunächst im Rahmen des HBM4EU-Projekts angewendet, können aber auch für Regulierungsbehörden und Risikobewerter außerhalb dieses Projekts von Nutzen sein. Derzeit sind HBM-GVs für Phthalate [7], Bisphenol A und S [8, 9], Cadmium [10] und Aprotische Lösemittel [11] veröffentlicht.

In jedem Land wurden nationale Gremien eingerichtet, um die Aktivitäten zu koordinieren und so eine solide Plattform für das Human-Biomonitoring auf gesamteuropäischer Ebene zu schaffen. Diese sogenannten National Hubs sollten die HBM-Expertise des jeweiligen Landes bündeln und nationale Anforderungen über die nationale Kontaktstelle in das Projekt einfließen lassen, umgekehrt aber auch von den Ergebnissen aus HBM4EU profitieren. Der deutsche National Hub bestand aus der HBM-Kommission sowie Vertreter:innen der deutschen HBM4EU-Partner und weiterer Behörden.

## Aligned Studies und Datenverfügbarkeit

Im Rahmen der sogenannten HBM4EU Aligned Studies wurden HBM-Studien aufeinander abgestimmt und zusammengeführt. Die Aligned Studies sind eine Erhebung, die darauf abzielt, möglichst harmonisierte HBM-Proben und -Daten aus (nationalen) Studien zu sammeln, um interne Expositionsdaten abzuleiten, die für die europäische Bevölkerung über eine geografische Verteilung repräsentativ sind. Die Sammlung der aktuellen Expositionsdaten von EU-Bürger:innen erfasst dabei 3 Altersgruppen: Kinder (6–11 Jahre), Teenager (12–19 Jahre) und Erwachsene (20–39 Jahre). Je nach Altersgruppe wurde die körperliche Belastung mit den priorisierten Substanzen bewertet. Die Daten zur Belastung der Menschen mit Umweltchemikalien aus dieser groß angelegten Studie tragen zur Überprüfung bestehender Regulierungen und zur Weiterentwicklung der Umwelt- und Chemikalienpolitik bei [12, 13].

Die wissenschaftlichen Artikel zu den Belastungsdaten der priorisierten Substanzen werden innerhalb der nächsten Monate veröffentlicht. Allerdings können bereits jetzt die Belastungsdaten im HBM4EU-Dashboard angeschaut werden.

## Veröffentlichung der Daten über IPCHEM und das HBM4EU-Dashboard

Die im Rahmen von HBM4EU verwendeten und erhobenen Daten werden über die Informationsplattform für die Überwachung von Chemikalien (IPCHEM)^2^ zugänglich gemacht.

Weiterhin werden die Daten, die unter HBM4EU erhoben wurden, mit Hilfe des European Human Biomonitoring Dashboard (EU HBM-Dashboard)^3^ einer breiteren Öffentlichkeit vorgestellt. Das Dashboard ermöglicht die Visualisierung von zusammenfassenden Statistiken aus den bestehenden HBM4EU-Datensammlungen, so dass Expositionsniveaus und Trends der chemischen Belastung der europäischen Bevölkerung untersucht werden können. Es werden auch Informationen zu Nachweisgrenzen (LOD) bzw. Bestimmungsgrenzen (LOQ) der Analysemethoden gegeben. Außerdem gibt es eine Filterfunktion, mit der HBM-Ergebnisse getrennt nach Geschlecht, Alter, Bildungsgrad usw. angezeigt werden können. Die aktuellen Daten sind auf die 18 prioritären HBM4EU-Stoffe beschränkt. Die im Dashboard enthaltenen Daten wurden auf standardisierte und vergleichbare Weise erhoben oder nach bereits früherer Erhebung postharmonisiert.

## Belastungsdaten zu den priorisierten Substanzen

Insgesamt zeigen die Ergebnisse, dass die Belastungen der EU-Bevölkerung mit den priorisierten Substanzen oftmals zu hoch sind und weiterhin Handlungsbedarf seitens der Politik besteht. Als Beispiele seien genannt:

### Phthalate.

Bedenklich hohe Belastungen mit bereits stark regulierten Phthalaten wurden in der europäischen Bevölkerung nachgewiesen. Alle untersuchten Kinder und Jugendlichen waren mit reproduktionstoxischen Phthalaten belastet. Es konnte zwar eine Abnahme der mittleren Belastung mit regulierten Phthalaten beobachtet werden, die Gesamtbelastung von ca. 17 % ist allerdings immer noch zu hoch. Gleichzeitig wurde ein Anstieg der Belastung mit Phthalatersatzstoffen beobachtet, deren langfristige gesundheitliche Auswirkungen derzeit wenig erforscht sind.

### Perfluorierte Alkylsubstanzen (PFAS).

PFAS wurden im Blut aller untersuchten Jugendlichen aus Europa nachgewiesen. Bis zu ein Viertel der Jugendlichen ist mit Konzentrationen belastet, bei denen eine gesundheitliche Beeinträchtigung nicht mehr mit ausreichender Sicherheit ausgeschlossen werden kann. Vorwiegend gehen die Belastungen von bereits verbotenen, jedoch äußerst langlebigen Verbindungen aus.

### Mischungen.

Es wurde nachgewiesen, dass unsere Körper mit einer Vielzahl von Industriechemikalien belastet sind und ein Wechsel des bestehenden Regulierungsparadigmas hin zu der Betrachtung von Mischungseffekten dringend erforderlich ist.

## Bürgerbeteiligung und öffentlich verfügbare Materialien

Im Rahmen von HBM4EU wurden sogenannte Fokusgruppendiskussionen durchgeführt, bei denen mit Bürger:innen über die Wahrnehmung der Umweltbelastung mit Chemikalien gesprochen wurde. Die Ergebnisse zeigen, dass die europäischen Bürger:innen sehr besorgt über die Lebensmittelsicherheit und die Umwelt sind [14].

Eine immer wichtigere Rolle nimmt bei heutigen Forschungsprojekten die Wissenschaftskommunikation ein. Zur bestmöglichen Nutzung der Ergebnisse wurde der HBM4EU Knowledge Hub ins Leben gerufen, welcher das Bindeglied zwischen Wissenschaft und Politik bildet.

Von Anfang an war das Ziel, die HBM4EU-Ergebnisse einem breiten Spektrum potenzieller Nutzer:innen auf kohärente, gezielte und zeitnahe Weise zu vermitteln und dabei die neuesten technologischen Entwicklungen zu nutzen.

Ein Hauptinstrument der Informationsbereitstellung ist die HBM4EU-Online-Bibliothek^4^, welche über die Website zugänglich ist. Sie bietet Zugang zu Leitlinien, Methoden, Protokollen und Forschungsergebnissen. Dazu gehören sowohl Protokolle, die im Rahmen von HBM4EU entwickelt wurden, als auch andere relevante, öffentlich verfügbare Anleitungen und Materialien.

Sämtliche Publikationen^5^ sind frei verfügbar. Zusätzlich zu den klassischen Formaten wurde eine Reihe von weiteren Informationsmaterialien entwickelt, welche kostenlos auf der Website abgerufen werden können:

### Policy Briefs.

Kurzdarstellungen (Toxizität, Exposition und Regulierung) der priorisierten Substanzen samt politikrelevanter Synthese der Gesamtergebnisse. Die Kurzdarstellungen sind auf spezifische politische Prozesse auf europäischer Ebene und in den Mitgliedstaaten ausgerichtet, einschließlich der europäischen Chemikalienverordnung REACH und relevanter sektoraler Politiken.

### Research Briefs.

Forschungsberichte zur Information von Wissenschaftler:innen und politischen Entscheidungsträger:innen mit aktuellen Informationen zu bestimmten Bereichen des HBM4EU-Projekts.

### Deliverables.

Berichte zu den einzelnen Projektergebnissen.

Speziell an die allgemeine Bevölkerung gerichtet sind die *HBM4EU-Factsheets*, die *Infografiken* und die HBM4EU-*Erklärvideos*, welche auf der Website in der Rubrik „Citizen’s Corner“^6^ abgerufen werden können.

## Fazit

Der vorliegende Artikel ist nur ein kleiner Ausschnitt aus dem Spektrum von HBM4EU. Weitere Arbeitsschwerpunkte lagen in der Verknüpfung von Exposition und gesundheitlichen Aspekten oder beschäftigten sich z. B. mit der Entwicklung von neuen Nachweismethoden und Expositionsmodellen. Eine vollständige Darstellung der einzelnen Forschungsbereiche findet sich auf der Projektwebsite www.hbm4eu.eu.

Die Ergebnisse des Projekts zeigen deutlich, dass für den Großteil der prioritären Stoffe und Stoffgruppen bei einem Teil der Menschen in Europa die Belastung so hoch ist, dass entweder gesundheitliche Wirkungen nicht mehr mit ausreichender Sicherheit ausgeschlossen werden können oder durch die inneren Belastungen ein zusätzliches Krebsrisiko besteht. Mit den harmonisierten und auf höchstem Niveau qualitätsgesicherten Analysedaten liegen nun erstmals belastbare Informationen über die tatsächliche innere Belastung der Menschen in Europa und deren Unterschiedlichkeit in den einzelnen Ländern vor.

HBM4EU hat den Nachweis geführt, dass im Bereich der Chemikalienpolitik noch Verbesserungsbedarf besteht. Die HBM4EU-Daten beschreiben den aktuellen Belastungsstand, gegen den künftig die Erfolge der kürzlich festgelegten EU-Strategien im Rahmen des europäischen „Grünen Deals“ (Green Deal) gemessen werden können. Zu diesen Strategien, die dem besseren Schutz vor unerwünschten Chemikalienwirkungen dienen, gehören beispielsweise der „Null-Schadstoff-Aktionsplan“ (Zero Pollution Action Plan), die „EU-Chemikalienstrategie für Nachhaltigkeit“ (Chemical Strategy for Sustainability) und die „Vom-Hof-auf-den-Tisch-Strategie“ (Farm to Fork Strategy).

Die Hauptergebnisse des Projekts, wie zum Beispiel die Belastungsdaten der EU-Bevölkerung und deren toxikologisch-gesundheitliche Bewertung, werden in Form eines HBM4EU – Special Issue im November 2022 veröffentlicht werden.

## Footnotes

1 https://www.hbm4eu.eu/hbm4eu-substances/hbm4eu-priority-substances/. Zugegriffen am 25.07.2022.

2 https://ipchem.jrc.ec.europa.eu/. Zugegriffen am 25.07.2022.

3 https://www.hbm4eu.eu/what-we-do/european-hbm-platform/eu-hbm-dashboard/. Zugegriffen am 25.07.2022.

4 http://www.hbm4eu.eu/library/. Zugegriffen am 25.07.2022.

5 https://www.hbm4eu.eu/result/publications-2/. Zugegriffen am 25.07.2022.

6 https://www.hbm4eu.eu/citizens-corner/. Zugegriffen am 25.07.2022.
